# Calcified Leiomyomata Presenting as Recurrent Pregnancy Loss

**DOI:** 10.1155/2020/2921472

**Published:** 2020-08-18

**Authors:** Mohammad Marwan Alhalabi, Ameer Kakaje, Marwan Alhalabi

**Affiliations:** ^1^Faculty of Medicine, Damascus University, Damascus, Syria; ^2^Department of Reproductive Medicine, Genetics and Embryology, Damascus University, Damascus, Syria

## Abstract

Recurrent spontaneous abortion (RSA) is a problem that faces women for a variety of reasons. Although leiomyomata is relatively common, calcified leiomyomata which is called “womb stones” is a very rare cause of RSA. These womb stones are correlated with retained products from conception and osseous metaplasia. We report a very rare case of a large calcified leiomyomata which caused secondary infertility and pregnancy loss of 7 pregnancies due to spontaneous abortions.

## 1. Introduction

Recurrent spontaneous abortion (RSA) occurs in approximately 1% of females in the reproductive age and is of a wide variety in nomenclature (recurrent pregnancy loss, recurrent miscarriage) [1]. RSA is defined as two or more of pregnancy losses according to the American Society for Reproductive Medicine (ASRM) [2].

Calcification in the endometrium of the uterus is not very common in infertility cases, and it has a correlation with the products retained from conception and with osseous metaplasia. Ultrasonography is the most reliable method of diagnosis for endometrial calcifications [3]. Infertility and recurrent abortion are uncommonly caused by the calcific endometritis [4]. The existence of bone-like tissue within the uterine cavity is called endometrial osseous metaplasia and affects about 0.15% of the patients referred to obstetrics and gynecology clinics [5].

The term “womb stones” refers to calcified appearance of leiomyomata in radiological imaging [6–8]; such osseous endometrial metaplasia is also of a wide nomenclature (uterine bone, womb stones, and calcified fibroid) [4, 9, 10]. We present herein a case of a large calcified leiomyomata (womb stone) suspected to be the cause of secondary infertility and idiopathic pregnancy loss of 7 consecutive spontaneous abortions.

## 2. Case Report

**Figure 1:**
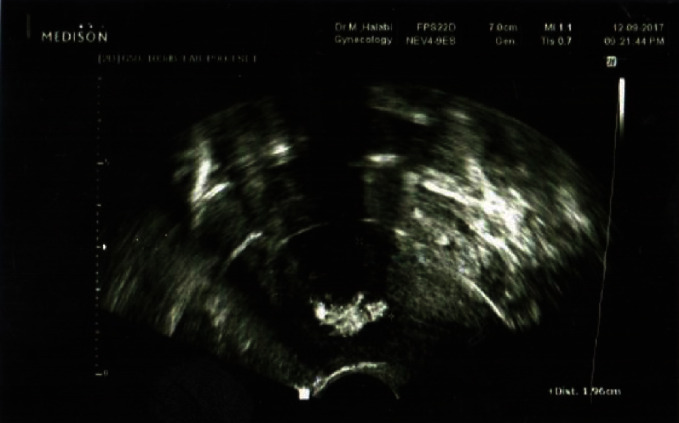
View of the uterus during abdominal ultrasonography.

A married couple presented to our fertility clinic complaining of recurrent pregnancy loss for ten years. The wife was 32 years old and had pregnancy loss for seven times occurring in the second trimester in each pregnancy with the last one being a year from the date of the clinic visit. In each pregnancy, her pregnancies were monitored by an obstetrician and the loss of pregnancy was verified by ultrasonography in each case. There was no history of primary infertility or any delay of conception. Menstrual cycles were regular with no dysmenorrhea or any other symptoms. Gynecological physical examination was unremarkable, and the uterus was normal in size, contour, and no tenderness was noted. It was also anteverted and anteflexed, all adnexae were normal, and hormonal and chemical parameters were also within normal range. The medical history was otherwise unremarkable. She had a surgical history of dilation and curettage (D&C) and an open ovarian cystectomy. An abdominal ultrasonography was conducted and showed a normal anatomy of the uterus with subendometrial calcifications in the frontal wall of the uterus, spanning 2 cm in diameter (Figure 1).

**Figure 2:**
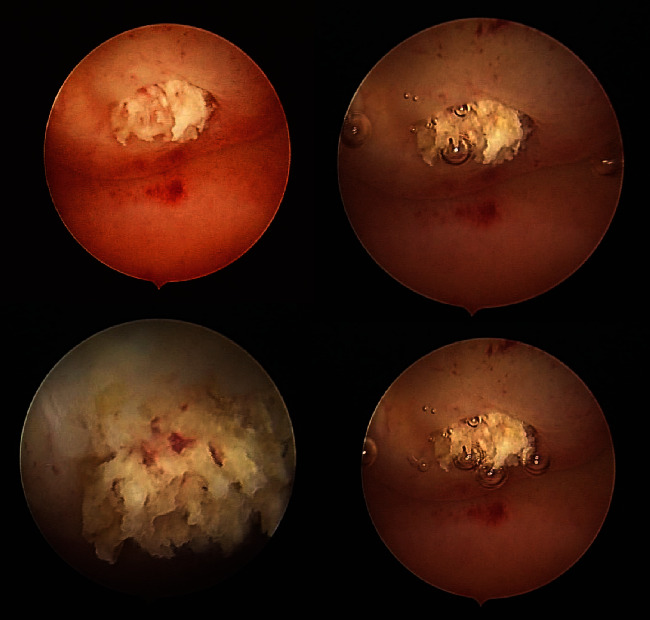
The large calcification shown during hysteroscopy.

**Figure 3:**
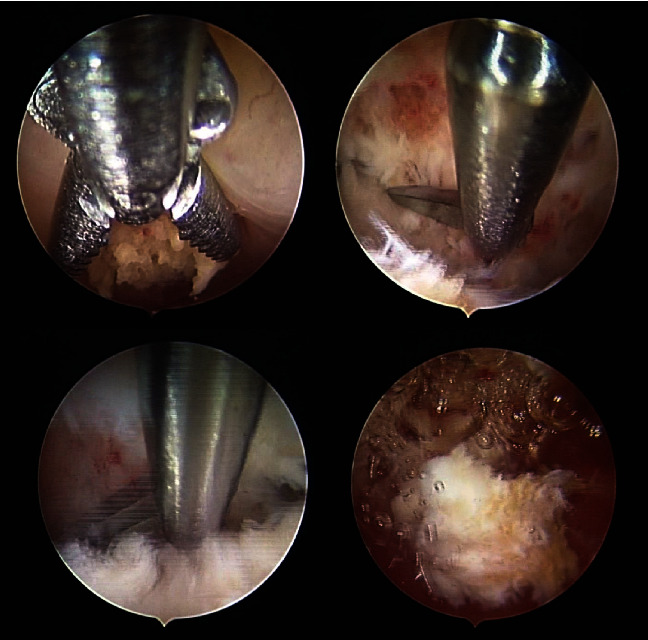
Showing the removal of the uterine bone through hysteroscopy.

**Figure 4:**
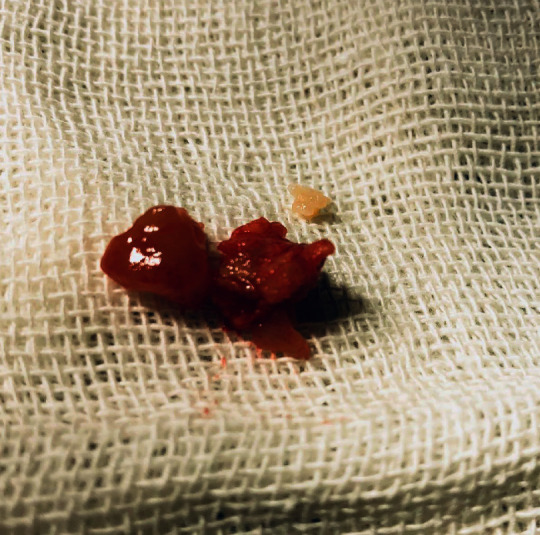
The gross view of the large calcification after removal.

**Figure 5:**
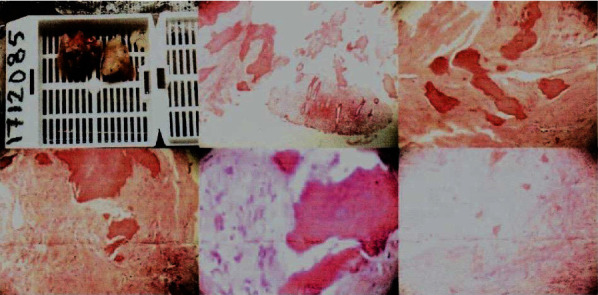
The microscopic pathological view of the calcification.

A later abdominal ultrasonography showed normal ovulation with both ovaries showing as normal in bulk, echotexture, and follicle maturation, and the calcified endometrium was also seen. The patient underwent hysterosalpingogram which showed partial obstruction of the fallopian tubes. The patient was referred to conduct hysteroscopy for diagnosis and treatment. Hysteroscopy showed a large calcification with multiple calcific protrusions in the endometrium (Figure 2), and it was removed (Figure 3), and everything else was within normal limits. The calcification was sent to pathological analysis which concluded a leiomyoma with calcifications and no malignancy (Figures 4 and 5).

## 3. Discussion

Endometrial calcification is an uncommon finding, and the presence of uterine bone tissue has been reported in several reports in the past years [11–23], and the affected couples may have already suffered great emotional trauma and stress. One frequently mentioned theory about the formation of uterine bones is the fetal bone retention after miscarriage or pregnancy termination [11, 12, 16, 24–28], but this requires at least one previous 12-week-long pregnancy having the embryo reach endochondral ossification [11, 15, 24]. It was reported that osseous metaplasia usually presents itself as diffuse, sporadic ossification in the uterine endometrium without a reaction in the tissues around a retained fetus [13, 24]. Osseous metaplasia is also associated with uterine bone formation and is correlated with chronic inflammation and destruction of endometrial tissue which can cause endometrial stromal cell heteroplasia into bony tissue [13–15, 24]. Furthermore, chronic endometritis causes superoxides and tumor necrosis factor to be released, and the exposure in the long term negatively affects stromal cells especially in patients with superoxide dismutase deficiency [14]. Another theory suggests that there are retained embryonic cells without a previous pregnancy which underwent heteroplasia into bony tissue; this theory can also explain the presence of womb stone [29]. Although in our study the patient did not complain of secondary infertility, the patient complained of recurrent pregnancy loss of all previous pregnancies, and therefore, the uterine bone was not suspected to be caused by fetal bone retention and was more suspected to be caused by the theory of endometrial fibroid calcifying metaplasia, and the pathological report of the specimen obtained concluded the specimen to be a calcified endometrial leiomyoma.

Calcific endometritis has also been correlated with Asherman's syndrome [4, 9]. However, in our patient, despite having a history of D&C, the patient did not complain of any of the symptoms of endometritis or Asherman's syndrome.

Calcification of the endometrium has also been reported without a history of an intrauterine device (IUD) or malignancy [9, 10]. Osseous metaplasia of the endometrium is less commonly reported than uterine bone [4, 9, 10], and only few cases reported spontaneous abortion to be related to endometrial ossification [21]. ^∗∗∗∗^Endometrial ossification was described to cause many gynecological disturbances such as symptoms of menorrhagia, metromenorrhagia, abnormal vaginal discharge, pain in the pelvis, and bone fragments passing through the vagina spontaneously [3, 12, 13, 16, 22–28]. Several cases reported that normal conception was achieved after repeat curettage or hysteroscopy of the uterine endometrium which led to removal of the ossification of the endometrium [3, 12, 13, 21–23, 25, 28], so we speculate that endometrial bone does permit the implantation of the embryo but prevents pregnancy from being sustained. It was also reported that uterine bone can act as an intrauterine contraceptive device causing failure of implantation [12]. It has been indicated by almost all previous publications that abnormal endometrial tissue has been handled by surgical removal [3, 11–14, 17, 19, 22–26, 30–33]. The removal of abnormal endometrial tissue either by D&C or hysteroscopy has also been reported in order to treat intrauterine adhesions through metroplasty and resection of submucous fibroids, therefore resolving problems relating to infertility and recurrent pregnancy loss [3, 12–14, 22, 23, 25, 28, 31, 32].

## Data Availability

Data will be made available upon reasonable request.
